# Women’s Experiences with Oral and Vaginal Pre-Exposure Prophylaxis: The VOICE-C Qualitative Study in Johannesburg, South Africa

**DOI:** 10.1371/journal.pone.0089118

**Published:** 2014-02-21

**Authors:** Ariane van der Straten, Jonathan Stadler, Elizabeth Montgomery, Miriam Hartmann, Busiswe Magazi, Florence Mathebula, Katie Schwartz, Nicole Laborde, Lydia Soto-Torres

**Affiliations:** 1 Women’s Global Health Imperative, RTI International, San Francisco, California, United States of America; 2 Center for AIDS Prevention Studies, Department of Medicine, UCSF, San Francisco, California, United States of America; 3 Wits Reproductive Health and HIV Institute, School of Clinical Medicine, University of the Witwatersrand, Johannesburg, South Africa; 4 FHI 360, Research Triangle Park, North Carolina, United States of America; 5 Division of AIDS, National Institute of Allergy and Infectious Diseases/National Institutes of Health, Bethesda, Maryland, United States of America; Commissariat a l′Energie Atomique(cea), France

## Abstract

**Background:**

In VOICE, a multisite HIV pre-exposure prophylaxis (PrEP) trial, plasma drug levels pointed to widespread product nonuse, despite high adherence estimated by self-reports and clinic product counts. Using a socio-ecological framework (SEF), we explored socio-cultural and contextual factors that influenced participants’ experience of daily vaginal gel and oral tablet regimens in VOICE.

**Methods:**

In Johannesburg, a qualitative ancillary study was concurrently conducted among randomly selected VOICE participants assigned to in-depth interviews (n = 41), serial ethnographic interviews (n = 21), or focus group discussions (n = 40). Audiotaped interviews were transcribed, translated, and coded thematically for analysis.

**Results:**

Of the 102 participants, the mean age was 27 years, and 96% had a primary sex partner with whom 43% cohabitated. Few women reported lasting nonuse, which they typically attributed to missed visits, lack of product replenishments, and family-related travel or work. Women acknowledged occasionally skipping or mistiming doses because they forgot, were busy, felt lazy or bored, feared or experienced side effects. However, nearly all knew or heard of other study participants who did not use products daily. Three overarching themes emerged from further analyses: ambivalence toward research, preserving a healthy status, and managing social relationships. These themes highlighted the profound and complex meanings associated with participating in a blinded HIV PrEP trial and taking antiretroviral-based products. The unknown efficacy of products, their connection with HIV infection, challenges with daily regimen given social risks, lack of support–from partners and significant others–and the relationship tradeoffs entailed by using the products appear to discourage adequate product use.

**Conclusions:**

Personal acknowledgment of product nonuse was challenging. This qualitative inquiry highlighted key influences at all SEF levels that shaped women’s perceptions of trial participation and experiences with investigational products. Whether these impacted women’s behaviors and may have contributed to ineffective trial results warrants further investigation.

## Introduction

The identification of safe and effective HIV prevention options for women has been an ongoing public health challenge. To date, four trials of oral pre-exposure prophylaxis (PrEP) have reported effectiveness among men and women [1]–[4], and only one trial has reported modest but significant protection with pericoital dosing of a vaginal gel [5]. However, two PrEP trials with daily dosing, tested among female-only study populations, were unable to demonstrate effectiveness [6], [7]. Product adherence is considered to be a primary reason for the widely divergent effectiveness levels in these PrEP studies [8], [9]. The Vaginal and Oral Interventions to Control the Epidemic (VOICE) trial, which tested daily application of 1% tenofovir gel alongside daily oral tenofovir and tenofovir-emtricitabine (Truvada), showed no effectiveness for the three products tested among 5,029 women at 15 sites in Sub-Saharan Africa. Plasma drug levels point to widespread product nonuse despite high end-of-study retention rates and high self-reported product use [7].

Evidence from qualitative and quantitative data captured in previous microbicide studies suggests that women’s product acceptance and adherence is influenced by a wide range of proximate-level individual factors [10]–[12] such as preferences for product characteristics (e.g., gel consistency) [13], [14]; partner and relationship factors [15]–[17]; and broader contextual factors such as gender roles [18], [19], vaginal practices [20], [21], and social acceptance of product use and trial participation [22]. This literature also points to women’s active engagement in redefining microbicides as technologies that rejuvenate and cleanse the body of impurities and that enhance intimacy with their sexual partners [23]. Adherence to oral PrEP was reinforced by supportive spouses among serodiscordant couples [24]. Altruistic sentiments and other trial-related factors eased oral PrEP use among high-risk individuals, while social risk (including stigma), product characteristics, and side effects had the opposite influence [25].

**Figure 1:**
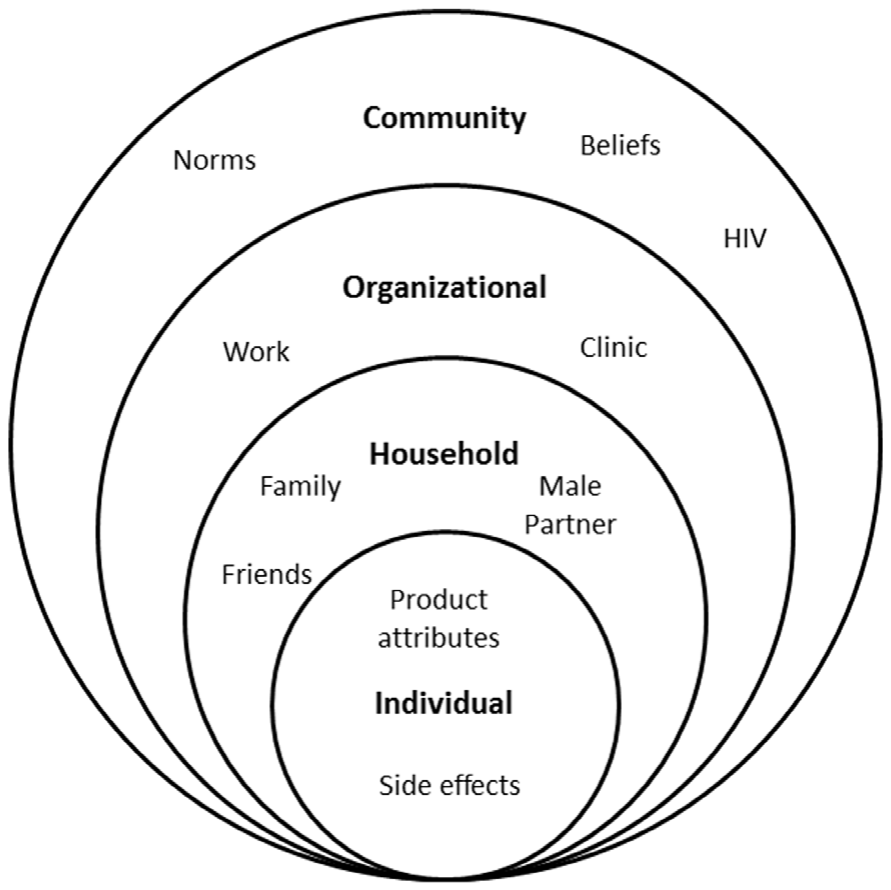
Socio-ecological Model of Factors Affecting Adherence in VOICE, and Levels of Influence.

Social science is increasingly recognized and included as a critical component of randomized controlled trials [18], [26]–[32]. The VOICE-C ancillary study design was informed by a socio-ecological framework (SEF) (Figure 1), which positions individual behavior within a broader context of influences at the household, organizational, and community levels. This model has been adapted to different areas of HIV investigation, including studies on risk behaviors, HIV vaccines, uptake of prevention of mother-to-child transmission services, and treatment adherence [33]–[37].

The main objectives of the VOICE-C study were to explore the socio-cultural and contextual factors that influenced daily PrEP regimen in the VOICE trial, to determine whether these factors differed between participants randomized to gels versus tablets, and to better understand women’s perceptions of and experience with investigational product use.

## Methods

### Study Design

VOICE-C was a qualitative exploratory ancillary study to the Microbicides Trial Network (MTN) VOICE trial, conducted at the Wits Reproductive Health Institute (Wits RHI), in Johannesburg, South Africa. The VOICE-C study took place concurrently with the parent VOICE study, between July 2010 and August 2012. It included four groups: VOICE participants (N = 102), male partners (N = 22), community advisory board (CAB) members (N = 17), and community stakeholders (N = 23). This paper focuses on the VOICE participants only, who were randomly preselected and assigned to one of three complementary VOICE-C interview modalities, chosen to offer a way to triangulate and strengthen findings: in-depth interview (IDI), serial ethnographic interviews (EI) [38], or an exit focus group discussion (FGD) [39]. Findings from other study groups will be presented in additional publications.

The VOICE trial was a phase IIB, double-blind, five-arm randomized, placebo-controlled PrEP trial evaluating the safety and effectiveness of once-daily oral tenofovir (TDF) and co-formulated TDF/FTC (Truvada) (tablet group) or once-daily vaginal tenofovir gel (gel group) for preventing HIV acquisition in 5,029 sexually active HIV-uninfected women, 18–45 years old at 15 sites in Uganda, Zimbabwe, and South Africa [7] (ClinicalTrials.gov NCT00705679). In the tablet group, eligible women were randomized to receive a once-daily dose of tenofovir tablet, Truvada tablet, or matching placebo in a 1∶1∶1 ratio. Because similarly appearing oral study products are not available for tenofovir, Truvada, and placebo, participants randomized to the tablet group took two tablets daily. Eligible women in the gel group were randomized 1∶1 to receive a once-daily dose of vaginal tenofovir gel or matching placebo. All VOICE participants also received free condoms, monthly HIV tests and risk reduction counseling, and yearly sexually transmitted infection diagnosis and treatment, as needed. Participants were followed for up to 36 months of study product use. At each monthly visit, they returned excess products and received a resupply along with product adherence counseling.

#### VOICE-C study setting and participants

**Figure 2:**
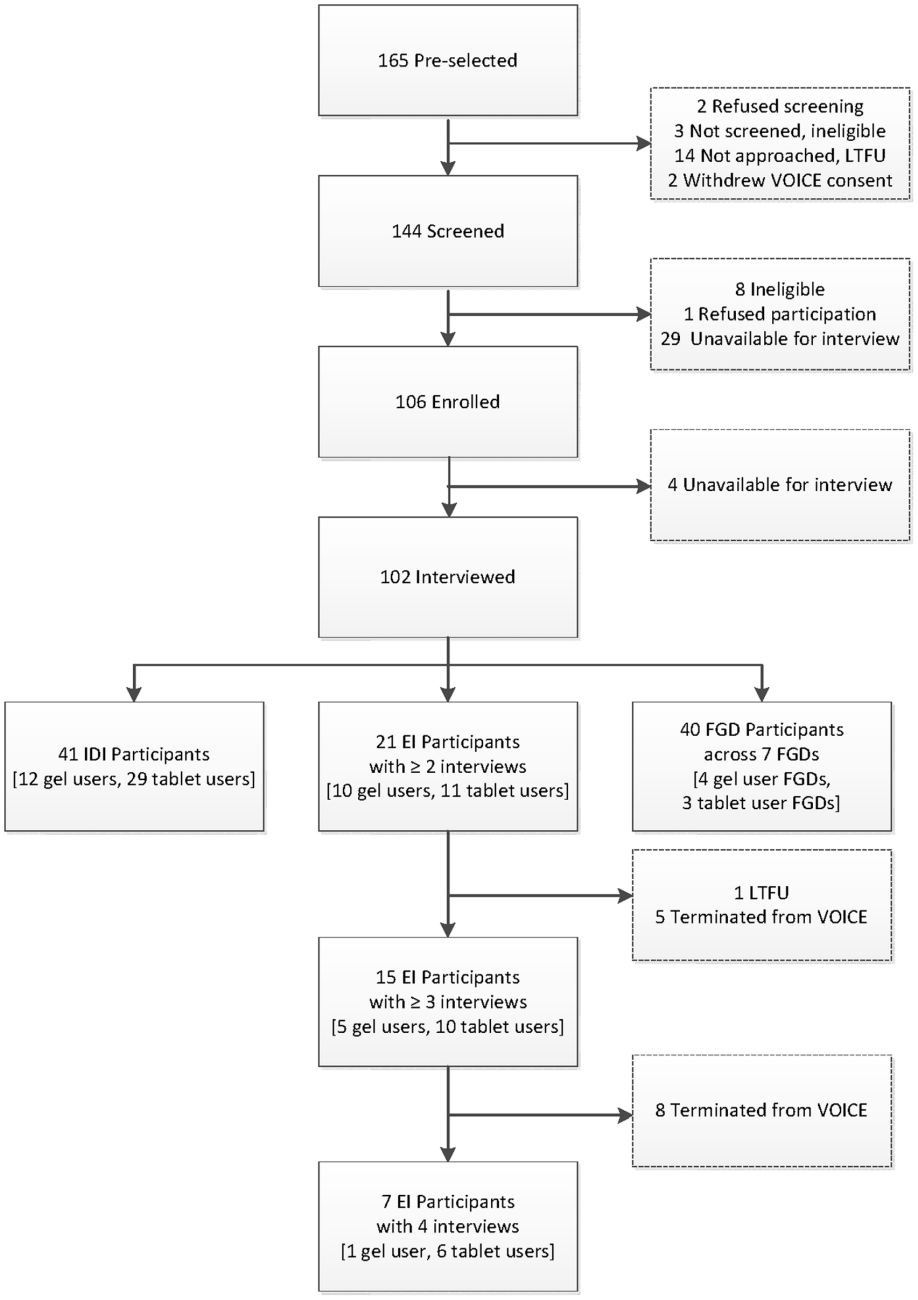
Female VOICE Participants Study Flow and Interview Mode. Legend: Approximately 150 women were targeted to be enrolled into VOICE-C ancillary study. A total of 165 VOICE participants were randomly preselected into VOICE-C (15 additional women were selected to accommodate for refusals and losses to follow-up within VOICE). A participant was classified as “enrolled” if she successfully underwent screening within the past 2 weeks and provided written informed consent for VOICE-C study participation. In most cases, informed consent took place on the day of the (first) interview. There were 144 women screened, 106 enrolled, and 102 interviewed. This represents 68% of the original target of ∼150 VOICE participants to be interviewed. The primary reason for the smaller sample was that the FGD group sizes were lower than expected. The target number of approximately 90 women enrolled in FGD was based on an estimate of 8–10 women and 8 FGDs. In practice, only seven FGDs were conducted, and the mean number of women attending each group was 5.7. Note: EI = ethnographic interview. FGD = focus group discussion. IDI = in-depth interview. LTFU = lost to follow-up.

The study was located in Hillbrow, a low-income, densely populated inner-city suburb of Johannesburg, in which a diverse mix of South Africans and migrant populations resides. VOICE participants were recruited from Hillbrow, other neighborhoods, and more distant townships such as Orange Farm and Soweto. At the Wits RHI site 354 women were enrolled into the VOICE trial between July 2010 and August 2012. Following enrollment into VOICE, a randomly preselected subset of women was invited to participate in VOICE-C. Women were eligible to screen if they had reached their Month Three visit prior to VOICE-C enrollment. To balance expected study attrition, we oversampled and preselected 165 VOICE participants; 144 were screened and 102 were interviewed in VOICE-C, which represents our analytical sample (Figure 2). Women provided written informed consent prior to participation in VOICE-C, and those eligible were randomly assigned to one of three VOICE-C interview modalities in the following proportion (1∶1∶3): IDI, serial EIs, or an exit FGD. Women were excluded if they had discontinued study product use (per protocol) permanently or for >2 months by the time of their scheduled VOICE-C interview. This included women who HIV-seroconverted prior to the time of the IDI, FGD, or start of EI series.

### Procedures

#### Data collection and management

IDI and FGD participants had a single interview conducted at the research clinic site. EI participants received two to four interviews over one year, conducted at the woman’s home, the research site, or another private location of her choice. An EI group participant was considered “retained” if she had at least two EI visits; 100% retention of the EI cohort was achieved (Figure 2).

IDIs and FGDs covered the following topics: salient issues in participants’ life at the community, organizational, household levels, as well as relationships (friends, family and male partners) perceived to influence trial participation, product acceptability and adherence; risk taking behavior; vaginal practices; and product preferences. The EIs were more informal and covered broader discussions of the participant’s life context, including their personal history, sexual/relationship history, and household composition. By emphasizing the social and physical context of participant’s lives and their product use, EIs intended to provide insight into the ways in which these contexts shape adherence that may be missed during more formal FGDs and IDIs [38].

Trained female research staff members conducted all interviews, in the language of choice of the participants. Interviews were audio-recorded, transcribed, and translated into English. Every transcript was reviewed twice for quality control, first by local field staff and then by the VOICE-C data center (RTI/Women’s Global Health Imperative in the United States). All participants received a brief demographic interview.

#### Procedural changes resulting from VOICE Data Safety and Monitoring Board (DSMB) recommendations

In September 2011, the VOICE DSMB recommended that the TDF arm be discontinued for futility. In November 2011, a similar determination was made for the tenofovir gel, and the gel group (active and placebo arms) was also discontinued. Women assigned to the Truvada or placebo arms continued participation (taking one tablet daily instead of two) until planned VOICE trial exit in August 2012. The DSMB-instigated changes contributed to several women who were randomly preselected for VOICE-C participation receiving an earlier interview than anticipated, being reallocated to a different interview modality (n = 13), or stopping their serial EIs early (n = 12) (Figure 2, Table 1).

**Table 1:** Demographic Characteristics of VOICE-C Ancillary Study Participants.

At time of VOICE-C (first) interview	N = 102	Percent
Age (mean, range)	26.8 (19–40)	
Currently married	22	22
Has current primary sex partner	98	96
Length of relationship in years (mean, range)	5.5 (0.1–25)	
Currently living with primary sex partner	44	43
Parity (mean, range)	1.2 (0–4)	
Number of children takes care of (mean, range)	2.1 (0–7)	
Completed secondary school or more	69	68
Income status		
Does not earn an income	44	43
Formal employment	52	51
Self-employment	2	2
Other	4	4
Ethnic group		
Zulu	27	26
Xhosa	13	13
Sotho	19	19
Ndebele	26	25
Other*	17	17
Religion		
Christian	94	92
Muslim	0	0
Other/none	8	8
Regularly attends religious services (1+/week)	85	83
Current residence is “home”	30	29
Years lived in current residence (mean, range)	8.9 (0–39)	
History of involvement with HIV research/work	43	42
Randomization assignment		
In-depth interview	28	27
Ethnographic interview	24	24
Focus group discussion	50	49
Type of interviews received**		
In-depth interview	41	40
Ethnographic interview	21	21
Focus group discussion	40	39
Initial interview conducted prior to first DSMB	44	43

*Other ethnic groups: Kalanga = 1, Khalanga = 1, Nyanja = 1, Shona = 2, Swati = 1, Swazi = 1, Tsonga = 3, Tswana = 4, Venda = 3.

**The procedural changes resulting from VOICE Data Safety and Monitoring Board (DSMB) recommendations contributed to several women who were randomly preselected for VOICE-C participation receiving an earlier interview than anticipated, being reallocated to a different interview modality, or stopping their serial ethnographic interviews early.

#### Coding and analyses

All transcribed interviews were coded in Nvivo (version 9.0, Burlington, MA) by the analysis team. A codebook was iteratively developed and an acceptable level of intercoder reliability (ICR) was set at ≥80% coding agreement for a set of 14 key codes that were determined *a priori* as those representing the key topics of interest, consistent with the conceptual framework and study objectives. Throughout the analysis process, approximately 10% of the transcripts were double coded by two or more team members to monitor ICR.

Given the longitudinal design of the EIs [40], a distinct process was used to capture change over time and confirm reliability. Following coding of each set of EIs, a summary table was used to capture longitudinal information on key topics. Each completed table was reviewed by a second team member who simultaneously reviewed the transcripts to confirm the reliability of the tabulated information and reach consensus around the accuracy of data synthesis.

An inductive approach to thematic analysis was used in which key factors, levels of influence, and explanations for mechanisms or pathways of influence were derived from systematic review, reduction, and interpretation of the coded data [39]. Data corresponding to each type of influence were further analyzed to reveal recurring patterns or salient “themes” to enhance our understanding of women’s study product experiences.

Demographic data were tabulated in SAS (version 9.0, Cary, NC). Comparison between the VOICE and VOICE-C samples at the Wits RHI site used baseline demographic data collected at the VOICE enrolment visit. Chi square tests (for categorical variables) and t tests (for continuous variables) were used to identify differences at the alpha *p*<0.05 level (Table 2).

**Table 2:** Comparison Between Characteristics of VOICE-C and VOICE Participants at the Johannesburg Site (Wits RHI).

Characteristics	VOICE-C sample		Non-VOICE-C sample at Johannesburg site		p-value*
At time of VOICE trial enrolment	(N = 102)	%	(N = 252)	%	
Age	25, 25.9 (18,39)		25, 25.7 (18,40)		ns
Currently married	16	16	6	15	ns
Currently lives with primary sex partner	45	44	94	37	ns
Parity	1, 1.1 (0,4)		1, 1.2 (0,5)		ns
Ethnic group/tribe					
Zulu	29	28	63	25	
Xhosa	11	11	35	14	
Sotho	16	16	35	14	
Ndebele	28	27	65	26	
Other	18	18	54	21	
Completed secondary school or more	72	71	181	72	ns
Earns an income	33	32	88	35	ns
Number of rooms in current home	3, 2.9 (1,8)		3, 3.2 (1,16)		ns
Treatment assignment					ns
Truvada	22	22	49	19	
Gel placebo	19	19	52	21	
Oral placebo	22	22	49	19	
TDF	18	18	52	21	
TFV gel	21	21	50	20	
**During VOICE trial follow-up**					
Retained at product use exit visit	99	97	218	87	0.002
Adherence per returned clinic product count**	N = 102 0.99, 0.92 (0.15,1.19)		N = 248 0.91, 0.77 (0.01,1.29)		<.0001
Adherence per number of days in past week with product use (CRF)***	N = 91 100, 91.84 (0,100)		N = 189 100, 93.05 (0,100)		ns
Adherence per number of days in past week with product use (ACASI)***	N = 92 100, 78.73 (0,100)		N = 189 100, 85.64 (0,100)		ns

*Chi square of Fisher’s exact test for categorical variables, Wilcoxon Normal for continuous variables.

**Calculated as the ratio of the number of tablets or gel not returned over expected product use days cumulatively across all follow-up visits.

***Estimated at 3-month visit. Sample size is smaller because 60 participants missed the first quarterly visit; calculated as percentage of days in past week with self-reported product use.

Note: ACASI = audio computer-assisted self-interview. CRF = case report form. ns = non-significant. All continuous variable summaries are median, mean (minimum, maximum).

#### Ethical and regulatory approvals and study monitoring

The study protocol was reviewed and approved by the Institutional Review Boards at RTI International and the Human Research Ethics Committee of the University of the Witwatersrand, and overseen by the regulatory infrastructure of the National Institutes of Health and MTN. The study was monitored at approximately 6-month intervals by FHI 360 and RTI.

## Results

### VOICE-C Sample Characteristics and Product Adherence

Of the 102 VOICE participants interviewed in VOICE-C, 41 received one IDI, 21 completed two to four EIs, and 40 joined one of seven exit FGDs (Figure 2). Women’s mean age was 26.8 years and 96% currently had a primary sex partner although only 22% were married; 68% had completed secondary school; about half had formal employment. This sample was highly mobile with only 29% identifying their current residence in Johannesburg as “home” and reflected a large ethnic diversity (Table 1). VOICE-C participants had baseline characteristics similar to the other VOICE participants at the Johannesburg site (N = 252). However, VOICE-C participants were more likely to have been retained in the VOICE trial, and product adherence level was higher by some measures (e.g., clinic product counts), but not others (e.g., self-reports; see Table 2). Nevertheless, in a random subsample of VOICE participants (N = 60) assigned to active products at the Johannesburg site, 53% had no detectable drug in any plasma specimen tested, a finding similar to the overall VOICE study drug-level results [7]. This low level of drug detection did not differ significantly by VOICE-C participation status (data not shown).

### Qualitative Narratives About Product use and Nonuse

During VOICE-C, many women discussed the challenge of incorporating daily product use into their lives. However, few acknowledged lasting nonuse; when mentioned, it was mostly related to missed visits and lack of product replenishments, employment or school schedule, or out-of-town travel to visit family [41]. Typically, women acknowledged occasionally skipping a couple of doses or referred to missing the correct *time* for dosing when discussing usage problems. These situations were related to forgetting, feeling bored or lazy, or being busy or “on the go,” as Hildah and Phumzile (all names are pseudonyms) explained:

Ah, maybe once or twice you know if I am in a place whereby I didn’t expect to sleep and I ended up sleeping there then I would miss my tablets if I had forgotten them at home, you see (Hildah, Tablet, IDI).

Yes, it would be 2 days [missing]…. But when they gave us the gels they said you must count from eight and count 8 hours, and then you insert the gel, so sometimes at the end of that 8 hours I would be at school and I wouldn’t insert the gel at all (Phumzile, Gel, FGD).

Several women mentioned not inserting gel when visiting their partner if he complained about wetness. Tablet users mentioned skipping doses on weekends when socializing, or to avoid mixing tablets and alcohol, but most frequent was women’s dislike of side effects, anticipated or experienced. At trial completion, Hildah felt relieved to be “*done with the research*”; she was not taking the tablets regularly because she experienced several side effects that scared her, such as diarrhea and painful joints.

During repeated EIs, narratives elucidated more complex, and occasionally open discourses about nonuse. Several women acknowledged long-term challenges with daily use, which they were unwilling to discuss initially with the VOICE-C interviewers and which they did not disclose to VOICE clinical staff. This was exemplified by Thandi, who, during her first EI, described experiencing side effects for several weeks: feeling “*sick”* with gastro-intestinal problems after starting to take the study tablets. The side effects subsided as her body “*got used*” to the tablets. She recognized the importance of daily use and emphasized her motivation to help the research and to protect herself from a philandering husband, who was both unsupportive of her tablet use and occasionally violent toward her. She did sometimes “*forget*” to take the tablets, only to take them at a later time. By her third quarterly EI, however, Thandi acknowledged being “*bored*” by the daily tablets, and complained that they aggravated her heartburn. She claimed she still took the tablets, motivated in part by knowing her blood was tested for the drug. However she did it secretly, because her husband would forbid her. Nevertheless, she questioned the personal benefits of taking the tablets before they had been “*verified*,” or proven effective. At her final EI, Thandi conceded that she had not taken the tablets for some time because of dislike, boredom, and her heartburn:

Since I have been in the study I have learned something which is that not all of us are going to use the tablets even if we know that this is the only way to protect ourselves from getting HIV and AIDS, you know. Some of us will say we have forgotten to take the tablets, but that’s not an excuse every time to keep on saying you forgot to take the tablets, just tell the truth and say you are tired and don’t want to take the tablet (Thandi, Tablet, EI#4).

Thandi realized she had deviated from the study’s expectations and acknowledged failing to tell the clinic nurses about her nonuse, although she minimized this, saying she felt that other participants took their products properly so it would not affect the study results.

Like Thandi, a few other participants acknowledged lasting nonuse, although candid discussions were the exception rather than the rule in VOICE-C. Lerato described misrepresenting her product use to VOICE clinical staff. Importantly, she felt that such face-to-face misrepresentation may be acceptable because the study will ultimately know the truth from her blood results.

[The nurse] asked me if I had stopped taking the tablets, and I only said to her I skipped them for a single day on the 25th. Then that was it and that was the only time I missed the tablets.

Interviewer: What is it that made you not to tell her that you stopped taking the tablets?

I just thought of not telling her, hey. But it doesn’t matter as the [blood] results will be coming back [laughs] (Lerato, Tablet, EI #3).

All VOICE participants were informed that their blood was tested for the study drugs, and several, like Thandi, mentioned that the blood testing motivated product use while others, like Lerato, stated that use/nonuse would be detected through this biomarker.

Nonuse also figured prominently in women’s explanations of the futility results for tenofovir tablets and gel in September and November 2011. Nyaradzo explained her intense reaction to these results:

I felt sick because I was telling myself that it does work and even now I wish that…they can get another thing to prevent HIV. […] But I still feel that…it is just that we were not using it properly. That’s why they found that it is not effective. […] Because the thing is I also know that sometimes I didn’t insert it. Maybe another person also had a similar problem or a different one which makes them not to insert it every day (Nyaradzo, Gel, EI #2).

This was sometimes framed in moralistic terms with the products failing because of participants’ “*dishonesty*” (Karabo, Gel, FGD). Otillia also blamed the other participants:

We would sit and talk in the waiting area, others would be busy complaining and others would be saying that the tablets are boring them. […] These people don’t take the tablets at all times for them to stay in their circulatory system, I remember the last time we took blood tests, and at the clinic they said they wanted to see how much of these tablets were absorbed in our blood. I really think not taking the tablets at all is one of the reasons why we got these results (Otillia, Tablet, EI #3).

A few women offered insights into improved dosing and delivery strategy for PrEP, favoring a user-independent, long-acting approach, given women’s challenge with daily or coitally dependent products:

Women who are using contraceptive pills still fall pregnant. Some people get infected with HIV while condoms are available and the gels are being thrown away… So I think the injection is what a person cannot take out. The woman must just get injection because really, we do not listen (Neo, Gel, FGD).

In sum, VOICE-C participants acknowledged skipping doses and some candidly discussed lasting nonuse. However, most women maintained that they consistently used the study products, a finding clearly at odds with the biomarker evidence of overall low adherence in VOICE. Confronted with the trial’s futility findings, women stated widespread nonuse by the other participants.

### Narratives of Study Product Experiences

To better understand the context in which product nonuse occurred, we sought to further explore women’s *experiences* with the study products. By experiences we mean women’s knowledge, practice, and understanding of tablets and gel, irrespective of how much they were used. Recurrent topics emerged during analysis, which were combined into three overarching themes cutting across SEF levels, from household to community (Figure 1): ambivalence toward research, preserving a healthy status, and managing social relationships. These three themes highlighted the complexity associated with engaging in clinical research in general and participating in an HIV prevention trial, specifically.

#### Ambivalence toward research

Diverse ideas about research and researchers pervaded women’s narratives of product use in the VOICE trial. Women spoke of their role as active agents in the research process–in terms of their contribution to finding an effective HIV preventive. Generally, they liked the study clinic environment, valued the quality health care, and praised the research staff’s professionalism and support, despite lengthy visits and long study duration. One of the main reasons for joining the trial was to access health monitoring and quality care and services. Women mentioned that the educational sessions and counseling encouraged product use because these demonstrated staff’s continued concern and care about them. The care provided by the clinic built trust of the clinic staff and the products, despite rumors of intentional harm that participants were exposed to in the community. For example, Valencia, who had visited a government clinic when she was ill, said:

I am participating in the study and I trust it. Some nurses [at government clinics] asked questions and discouraged me. They said that they [the researchers] will infect you with sicknesses. If I wasn’t sure about the study I would have dropped out; because they said a lot of things about it (Valencia, Gel, FGD).

Like Valencia, other women’s narratives drew attention to a pervasive discourse that permeated the domestic, clinic, work, and neighborhood domains questioning the legitimacy of the trial, including reasons for targeting Black South Africans in medical research and pointed to the potential harm resulting from using experimental drugs. Furthermore, rumors about the exchange of blood for cash linked the trial to alleged satanic practices. The social effect of these discourses on product use is not easily established; however, unlike for Valencia, they seemed widespread enough to shake women’s trust in the research or their rationale for agreeing to be tested with investigational products when healthy:

You know it’s scary to hear that you will take tablets meant for HIV-positive people if you know very well that you don’t have it. So that won’t just be easy on you, even when you tell someone else that you are taking these kind of tablets they won’t understand and they will think that you are lying and you have the disease or maybe at the clinic they will infect you with it because they are using us to test and their question was why don’t they test this on animals? (Lilly, Gel, FGD).

Men’s resistance to their female partners’ trial participation was also a common theme. Family, household members, friends, and fellow participants occasionally tainted women’s perceptions of the products or undermined their trust in research. One woman said: “*So I sometimes think what if what my friends are saying is true, as they say ‘what if they are infecting you with AIDS using that gel?*’” (Phumzile, Gel, FGD).

Zanele, who attributed her weight gain to the gel, recalled a problematic conversation with fellow participants in the waiting room:

[That study] participant said, “Why don’t they do the research on themselves? They do it on us. I do not use that thing, I just put it there. I do not insert it. Why do they not use it? What if it causes a problem to us?” Then I said “Then what did you do? If you did not use [the gel] they will see that on your blood sample and at the end what do you say they must write?” She said, “I just said yes I used it every day” (Zanele, Gel, FGD).

Women’s accounts of their interactions within the clinic setting with fellow participants, and at home with household members and intimate partners, present multiple and often contradictory discourses of harm versus benefit, exploitation versus compliance, and individual needs versus communal concerns, all of which served to legitimize both use and nonuse of the trial products.

#### Preserving a healthy status

Participating in the VOICE trial was regarded as both healthy and risky. On the one hand, regular health screening and testing created a strong sense of well-being. On the other hand, women were aware of the potential for biological harm, given that the products were investigational, and their bodies may be exploited for scientific experimentation. Further, the knowledge that the study products contained antiretrovirals (ARV), which are used to treat AIDS, contradicted women’s construct of the healthy self that participation in the trial had created. Women wanted both to preserve their status of having good health and to be perceived as healthy by significant others. ARVs were viewed as medications “*for sick people*” (Thoko, Gel, EI #2) and using treatment for prevention was not well known–especially taking tablets for prevention. Community misunderstanding was widespread. As Frances said, “*the name ARV casts a shadow”* (Tablet, FGD).

Although many women mentioned their hope and belief that the study products were protective, there was widespread confusion and even disbelief as to why researchers would make HIV-negative participants take an ARV meant to treat HIV infection. This was stated earlier by Lilly and here by Thoko:

What I know is that ARVs are for people who are sick, why would they [researchers] give them to us even though we are not sick? I would not understand that because we are not sick (Thoko, Gel, EI #2).

Those who were not worried stated that as long as the drugs had no side effects or impact on their body, they were willing to take them. Indeed, ensuring one’s well-being seemed misaligned with taking potent medications daily and risking side effects, especially if these were noticeable (e.g., weight gain, skin complexion change) or were associated with sickness (e.g., diarrhea, vaginal discharge). Becoming plump, frequently mentioned as a side effect of the tablets, was perceived as a mark of being on ARV treatment for HIV-positive people. Living in tight quarters and with no separate toilet facility, Ida was concerned by the smell in her urine, which revealed her tablet use to her housemates and male partner. The problems caused by the trial products’ side effects were compounded by the lack of short-term personal benefit in taking the experimental drugs, until proven effective for prevention, as mentioned previously by Thandi, or here by Keneoe:

[People] would be able to use the products if they know that the products are working. They are going to use them; because now participants don’t know what effects the products have– that is why they miss their doses (Keneoe, Gel, EI #2).

Paradoxically, the side effects of products were also discussed in terms of potency and protection. For Rose, the side effects indicated that the tablets were protective against HIV infection:

[T]he tablets are also working because they have some reaction on us like some of us have headaches and become nauseous and stuff like that, so you would believe that means that these tablets have a certain possibility of reducing the risk of contracting HIV, you know [Rose, Tablet, FGD].

Monthly testing and repeated HIV-negative results created a sense of well-being, happiness, and safety. Several women said their products (gel or tablets) “*helped*” because they remained HIV negative even when not using condoms, and with a partner of unknown status or who refused to get tested:

I think [the tablets] help me because I cannot trust my partner and I don’t know whether he uses condoms since he does not want to use condoms with me. So he may also be tempted not to use condoms with another woman. Ever since I started to use these tablets I have been testing [HIV] negative (Nelly, Tablet, EI #1).

In the context of widespread product nonuse, these perceptions of protection are somewhat complicated to understand; however, they do highlight the premium placed by women on their own health.

Women used several strategies to reassert their healthy status and deal with frequent suspicions in their social entourage that they were infected because they were using ARVs. Strategies included selective disclosure, hiding the product containers, and discreet use so as not to raise unwelcomed questions or gossip. The gel boxes were bulkier, but fewer people knew what they were, whereas the tablet containers were recognized as ARVs and therefore had to be stored (or hidden) with greater care to avoid questioning. Among tablet users there was a greater fear of being pegged as HIV positive. Because of close living quarters, it was not always easy for participants to take their products unobserved. Tebogo displayed her tablet bottles to offset suspicion:

At first I was putting [the tablets] inside my bag and then I took them out of it and put them inside my wardrobe but then one of my friends opened my wardrobe. Because she saw that I was taking the tablets and she didn’t understand why I was taking the tablets even my partner didn’t understand why I was taking the tablets. So I put the tablets in open field so that they could understand that I was taking the tablets for the study and it’s not that I was sick or anything like that (Tebogo, Tablet, FGD).

When accused of hiding their HIV infection, some women took their suspicious relatives to a public testing center to prove they were HIV negative, or brought them to the study clinic for more explanation. Thereafter, some partners and family members became accepting and sometimes created a supportive atmosphere, such as helping to remind participants to use their product. In contrast, a few women experienced negative social consequences: one was discriminated against by her extended family who would not touch household objects she had touched, and another separated from her boyfriend who persisted in thinking she was HIV positive. Gladys recalled how her friend and fellow participant, who viewed herself as a hero when joining the study, eventually had to relocate because of her roommates’ discriminatory behavior:

I asked her how [her roommates] knew that she was drinking the tablets…. I asked her why she didn’t put her tablets in her wardrobe, and stuff like that. She was like, “I wasn’t ashamed of them because it’s a study, you know. I thought I was going to be a hero to say we have discovered this.” So, they despised her…there is that stigma, discrimination and stigmatization of those people who have got AIDS. So, they started to change the way in which they were living, you know. When we have drunk with this cup, they will just not touch it (Gladys, Tablet, EI #3).

Some study-related behavioral changes alerted others to product use and resulted in gossip as well. Angel’s friends noticed she had stopped drinking alcohol, and thus suspected she was on ARVs (Tablet, FGD). She also noted that alarms used as reminders alerted other people to tablet taking, which started gossip about her:

…like my family, I explained that I am attending a study but they don’t [believe] that I am attending a study, they just thinking I am HIV positive and I am hiding it. Now I am taking the tablets every day at 19∶00. When the phone alarm starts “tring tring” they then look at you and say: hey! and then I just look at them slightly and say [silence]… and then stand up. They say, “Okay, we are waiting.” Someone who is already drunk will tell you that, “Hey, get away, you are dying soon” (Angel, Tablet, FGD).

#### Managing social relationships

As evident in the narratives above, anticipated or experienced suspicion, questioning, discrimination, or misattribution of HIV-seropositivity within women’s social relationships influenced their experiences of the study products

Primary sex partners ranged in how they viewed or supported women’s product use and trial participation, from supportive to passive, unaware, or unsupportive. Those who were supportive provided money to get to the study clinic, reminded participants about product use, or allowed participants to use their cell phone alarm as a reminder. One woman indicated that because the gel did not alter sex, her husband was “*fine*” with her using it–and several others described that their partners did not “*complain*.” In other words, as long as the products did not interfere with their relationships, the male partners were likely to be perceived as passively accepting the products. Women who did not live with their partners (e.g., boyfriends versus husbands) were more likely to have partners unaware of product use.

For women who disclosed trial participation to partners, a commonly described challenge related to partners *“not understandin*g” the purpose of the study or of using the gel/tablets. In some cases partners came to understand and “*accept*” the products later [42]. Much of male partners’ lack of support or understanding was described in relation to their suspicion of the products–that “*these things will make you sick*” or make him sick. “*Sickness*” was a euphemism for larger fears about the products causing more severe harm, such as uterine cancer, infertility, “*damage to the womb*,” and HIV acquisition. Additionally, some women said their partners felt threatened by the products because they may encourage women’s promiscuity. Partners’ discontent indirectly influenced women’s willingness to use products, or more directly made use difficult and promoted clandestine use. The opportunity for freedom conferred by the study products challenged gender roles:

So there are many things that we go through as women. So it’s entirely up to you, how you will treat yourself, you know, since some of us don’t have husbands… Then it can be painful to have a husband that will always be watching your every move (laughs) you know some men can know before you tell them that you have tablets and are using them and from that they can try to enforce their authority and tell you that they are the heads of the house and such men don’t allow you to do your own things, instead they always want to control you (Lilly, Tablet, FGD).

Women discussed being at risk for HIV, mostly because of unfaithful partners. Although most women framed their motivation to join the VOICE trial in terms of checking their health and getting quality health care services, in many cases, perceived risk was also explicitly stated as a reason to enroll and use the study products:

Emotionally, I had this thing that at least there is something that we are trying which would help us, because as women we have problems at home as men would go out and cheat while you are sitting still in the house. So, that is the thing which was making me to always take the tablets, you see (Zama, Tablet, IDI).

However, when staying in the study or taking products was at the expense of losing important relationships or resources, the choice was obvious:

[My friend], she withdrew from the study because she did not want to lose her boyfriend. So she quit the study. So yes the influence from outside, wrong things that people say does have an impact. As I said, I am also not working. I stay with the father of my children. Sometimes a person will just be difficult and say “either you choose your study or you choose me.” And you are thinking, in town I have to pay the rent and children have to eat. So you will just quit from the study because you do not want to lose that person (Nogoli, Gel, FGD).

The majority of participants were recent arrivals in Johannesburg who kept regular contact with their relatives and families across South Africa and neighboring countries. Although disruption in routine from travelling sometimes affected use, those who remembered to carry the product with them typically said they managed to continue to take it when visiting family. However, even those denying challenges with product use during family-related travels were subject to questioning by family members, especially when the products were revealed to contain ARVs. Family members worried about the health effects of taking ARVs. A woman’s uncle thought the tablets were “*street drugs*,” while for another, kin thought the gel was witchcraft or magical medicine (*muti*) used to “*catch a man*.”

Because women were concerned by others’ judgment, trust was a recurring topic that came up around decisions of disclosure. Persons who were close kin or trustworthy were those to whom participants selectively disclosed. Disclosure was also used to avoid being talked about behind one’s back or to gain support, including within more formal working relationships. Lynn was able to attend her study visits, even when the restaurant where she worked was busy, because she had told her coworkers and boss. Women living in a supportive environment, such as understanding household and family members or partners, mentioned fewer problems with using their products. A handful gained adherence buddies–HIV-infected relatives or occasionally main partners who reminded them to take products.

Waiting room discussions among participants were common, and as mentioned above, seemed to influence women’s own product experience in several ways. All but two participants reported that they knew or overheard other participants discussing their lack of product use, and in several instances this was described as demotivating. Discussions that emphasized suspicion about the research and products were disparaging, as mentioned above by Zanele and here by Palesa.

It is not that easy to accept that I have to take the tablets at a specific time. So, obviously you want an easy way out. So, if I come here and hear participants saying that they skip taking the tablets and whatever else they say I will definitely be influenced (Palesa, Tablet, IDI).

Also, talk of negative aspects of product attributes prompted new participants to wish they were randomized to a different product; it scared or discouraged them to use their assigned products. For gel, negative aspects included vaginal wetness, leakage, and dislike by male partners. However, participants also discussed the positive attributes of the gel, remarking on improved sexual experiences or strategizing on finding the right time for gel use (e.g., to avoid the wetness during the day, or around sex). Among tablet users, most discussions appeared to focus on negative attributes such as tablet size, taste, and associated side effects.

## Discussion

This qualitative study conducted in Johannesburg, South Africa, explored factors influencing study product experiences among 102 female participants in VOICE, a multisite HIV PrEP trial that was unable to demonstrate oral or vaginal product effectiveness. One of the key findings of this study was that, similar to the quantitative behavioral data captured in the parent trial, few VOICE-C ancillary study participants openly disclosed lasting product nonuse during IDIs or FGDs. However, women attributed non-compliance to other participants; in fact all but 2 of the 102 women reported that they knew of women who did not use the products or overheard participants discussing nonuse. Further, women who participated in serial EIs more openly discussed personal adherence challenges during their follow-up interviews. This suggests that the rapport established with interviewers fostered greater openness in qualitative interviews, and possibly enhanced honesty.

Nevertheless, women gave several important insights into their lives and study product experiences that helped explain low adherence to the daily regimen. These specifically related to social relations, resources, and organizational and community contexts. The SEF, which framed our analysis, highlighted the way in which women’s personal experiences were shaped by broader considerations encompassed by three overarching themes: the institution of research, perceptions of healthy status, and influential relationships. These themes were often interwoven, exhibiting the complexity of factors influencing women’s perceptions and experiences of the study products.

Women had to negotiate multiple and sometimes contradictory roles given the diverging demands of various social relationships that occasionally competed with those of the VOICE trial. Further, in a context of limited access to health resources [43], receiving quality health care through the study clinic had to be balanced with the requirement to take investigational drugs of unknown immediate personal benefit, and at nontrivial social risk [25], [44]. As noted in previous clinical trials involving both microbicides [22], [45] and vaccines [46], rumors about the malicious intentions of researchers and the dangers of participating in research contributed to the complex range of product use experiences described here. Additionally, of particular salience in VOICE was the daily requirement to take ARVs–in the form of a gel or tablet–to fulfill the trial’s PrEP objectives. The ARVs were associated with sickness and placed women in uncomfortable social situations, whereby they had to prove to their partner or relatives that they were indeed HIV negative to offset suspicion. This also contributed to internal struggles about taking HIV treatment when healthy. The novelty of PrEP for HIV prevention combined with pervasive HIV-related stigma [47], [48] led to considerable fear–or actual experience–of being identified as HIV positive, more so among tablet than gel users. Thus, women had to reconcile their altruism and desire for prevention with the shame of being treated as diseased by uninformed people, or judged as naively exploited through the research.

These contradictions, combined with limited social support for product use from trusted ones, lack of familiarity with ARV-based prevention, rumors of widespread nonuse in the waiting room, reassurance from their monthly negative HIV test results, and no feedback on actual adherence levels during the trial, may account for the high rates of nonuse. Some women indicated that the lack of real-time monitoring allowed them to mislead the staff and not take their products. They played down their inflated self-reports knowing that blood tests would provide researchers with accurate assessments. Although narratives highlighted the critical importance of product adherence to get valid study results, several women minimized the consequences of their own behavior in the context of a large, blinded trial, counting on others for compliance. Furthermore, when faced with the futility results of tenofovir tablets and gel, many continued to diffuse their personal responsibility by pointing instead to the other participants who failed to comply.

This study has several limitations: it was conducted at only 1 of the 15 VOICE trial sites, and therefore findings may not be generalizable to other sites. Another multisite qualitative study (http://www.mtnstopshiv.org/studies/4493) is currently underway, to further understand the context and issues surrounding participants’ widespread product nonuse and misreporting during VOICE. Although the VOICE-C sample may appear small, it is larger than typical qualitative studies that focus on depth rather than breadth for analysis [49]. We used multiple qualitative methodologies–FGDs, IDIs, and prospective EIs–that allowed us to explore the same questions in multiple ways, deepening our examination. Also, we feel confident that we were able to reach theoretical saturation [50] because the same topics emerged during analysis through our complementary interviewing methodologies. Through random selection, we ensured that the VOICE-C participants were representative of the parent trial sample at the Johannesburg site on baseline characteristics. Women in VOICE-C had a higher retention rate than other VOICE participants, an unsurprising finding given that a large proportion were assigned to FGD, which occurred after their product use end visit.

VOICE-C was an exploratory study, and insights for future research, PrEP trials, and demonstration projects have emerged. First, as previously argued [23], clinical trials are more than biomedical enterprises to test new drugs: they are social phenomena that create new social relations within the household, the clinical trial setting, the local community, and translocally with donor organizations and research agencies. These social relations will shape and reshape local knowledge and the meaning of participation in clinical trials and of testing experimental drugs. Additionally, drugs are not mere active pharmaceutical ingredients, they are social innovations that require commensurability within the lives of their adopters and their social network [51], [52], whether the adopters are clinical trial participants or real world users. Social motivations [53] for PrEP must be increased and supported at all levels: clinic staff, peers (specifically fellow participants), family, friends, and sexual partners. This can be facilitated through disclosure to trusted individuals (not just sexual partners) [10], formalization of adherence buddies [54]–[56], and a favorable social environment at the study site, including regular group discussions or workshops with participants to explore common experiences that create peer support. Second, the social cost of joining trials or PrEP programs should be minimized by reducing visit burden, study procedures, waiting time, and demands on participants. Encouraging couples’ and male partners’ participation –even if minimal– into studies may facilitate acceptance and support of product use for women in stable partnerships, as was seen in Partners PrEP [57], [58]. Social rewards, especially immediate and tangible ones, should be evaluated as a possible means to promote engagement in research [59]. Women’s altruistic and personal health motivations to join trials and undergo monthly monitoring can be leveraged to facilitate persistence with healthy behavior once enrolled, including consistent product use. Third, the intervention benefit of incorporating robust adherence monitoring tools with minimum opportunities for manipulation should be evaluated. These can provide rapid feedback to participants and can be linked to actual outcomes (e.g., drug level) or to more proximal behavioral steps (e.g., correct product use, getting an adherence buddy, attending workshops). Finally, more investments should be made to increase community-wide understanding of ARV for prevention, and to mitigate pervasive HIV stigma, which hinders access to prevention services [60].

In conclusion, the research community needs to acknowledge, and continuously remind itself, that new HIV preventives will not be readily embraced just because they are needed. Only through a better understanding of the social and structural contexts in which these innovations are introduced can we perhaps facilitate the successful testing and adoption of efficacious bio-behavioral HIV prevention approaches that can be used by women.
